# Multidisciplinary Approach in Libman–Sacks Endocarditis

**DOI:** 10.1155/cric/5505094

**Published:** 2025-11-19

**Authors:** Vikyath Satish, Maisha Maliha, Shalom Rosenbaum, Vishakha Modak, Kuan-Yu Chi, Ephraim Leiderman

**Affiliations:** Department of Medicine, Jacobi Medical Center/Albert Einstein College of Medicine, Bronx, New York, USA

**Keywords:** antiphospholipid antibody syndrome, Libman–Sacks endocarditis, marantic endocarditis, mitral valve, nonbacterial endocarditis, systemic lupus erythematosus, transesophageal echocardiogram, transthoracic echocardiogram, valve vegetation

## Abstract

**Background:**

Libman–Sacks endocarditis is a noninfectious form of endocarditis associated with systemic lupus erythematosus (SLE), antiphospholipid antibody syndrome (APS), and solid malignancies.

**Case Presentation:**

A 20-year-old woman with no medical history presented with left-sided weakness and facial drooping. Laboratory tests were unremarkable except for thrombocytopenia and a prolonged activated partial thromboplastin time. Brain magnetic resonance imaging revealed an acute ischemic infarct in the bilateral frontal lobes. A transthoracic echocardiogram (TTE) and subsequent transesophageal echocardiogram (TEE) identified two mitral vegetations with regurgitation: the first located on the posterior leaflet with perforation (1.2 × 1.0 cm) and the second (1.0 × 0.9 cm) situated on the subvalvular apparatus of the anterior leaflet. The patient reported no signs of infection, and multiple blood cultures showed no bacterial growth. A rheumatological panel indicated SLE and APS, which resulted in a diagnosis of Libman–Sacks endocarditis. The patient received treatment with Coumadin and hydroxychloroquine, and a surgical resection of the vegetation was scheduled if the valvular lesions did not resolve within 1 month. However, a follow-up TTE demonstrated near-complete resolution of the masses.

**Conclusion:**

A stroke in a young individual should raise suspicion for Libman–Sacks endocarditis. Due to limited evidence regarding surgical versus conservative management, a multidisciplinary approach is recommended. This case illustrates the successful conservative management of two large vegetations.

## 1. Introduction

Libman–Sacks endocarditis (LSE) is a noninfectious endocarditis associated with systemic lupus erythematosus (SLE), antiphospholipid antibody syndrome (APS), and solid malignancies. While stroke is the most common manifestation of LSE, there is limited data regarding valvular interventions and their timing. Here, we present a case of a 20-year-old woman admitted for a stroke who was subsequently diagnosed with SLE and APS. The patient also exhibited two large vegetations on the mitral valve and was scheduled for valvular replacement surgery due to the size of the lesions and the significant risk of recurrent emboli and stroke. In the meantime, the patient was managed conservatively with anticoagulation, which completely resolved the vegetations prior to planned surgery. This case illustrates the successful conservative management of two large vegetations that met the criteria for surgical intervention. We aim to encourage a multidisciplinary approach through an “endocarditis team” in this niche population to discuss the benefits and risks of treatment options.

## 2. Case Presentation

A previously healthy 20-year-old woman presented to the emergency department with a sudden onset of left-sided weakness in her lower face, arm, and leg. On examination, she demonstrated left-sided motor weakness in both her upper and lower extremities, along with a left-sided facial droop with sparing of the forehead. A stroke code was activated, and a noncontrast computed tomography scan of the head revealed a focal white matter lesion with edema in the right frontal lobe. Blood tests revealed thrombocytopenia (84 × 10^9^/L) and a prolonged activated partial thromboplastin time (99.5 s, upper limit of normal 36.5 s), while other parameters remained within the normal range.

Based on the patient's clinical presentation and relatively young age, the differential diagnosis at this juncture remained extensive and encompassed ischemic stroke, APS secondary to an underlying autoimmune process accompanied by thrombotic emboli, primary central nervous system vasculitis, neurosarcoidosis, Behçet's vasculitis, vasculitis secondary to a primary systemic immune disorder, central nervous system vascular malformation, and seizures with Todd's paralysis.

Brain magnetic resonance imaging (MRI) and head and neck magnetic resonance angiography (MRA) were conducted to further describe these findings, which revealed scattered areas of bilateral cortical and subcortical infarction, most prominent in the right frontal region with contrast enhancement. It also demonstrated punctate hyperdensities suggestive of cortical petechial hemorrhages and leptomeningeal enhancement. This pattern was consistent with acute and subacute infarction attributed to an embolic or vasculitic etiology affecting the right frontal middle cerebral artery.

**Figure 1:**
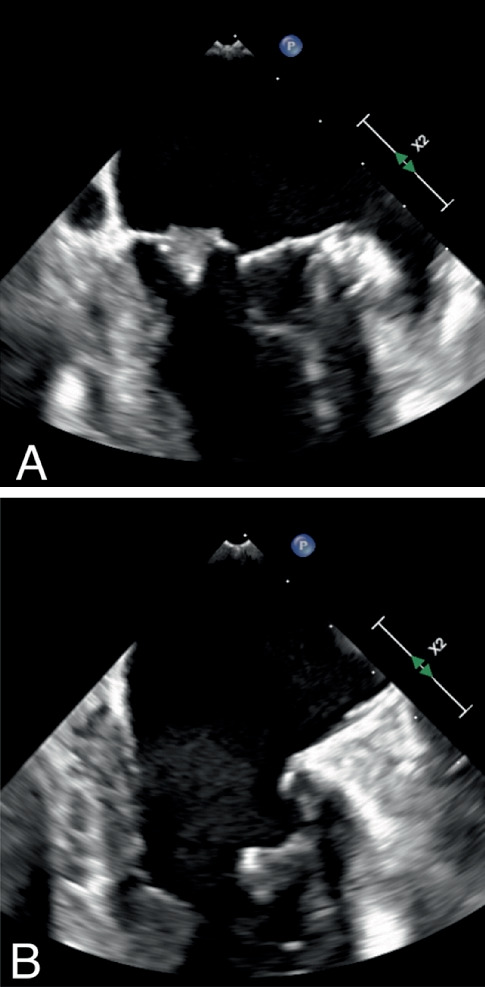
(A) Transesophageal echocardiogram demonstrating 1.2 × 1.0 cm vegetation on the posterior leaflet. (B) Transesophageal echocardiogram demonstrating 1.0 × 0.9 cm vegetation on the subvalvular apparatus of the anterior leaflet.

To explore the etiology of the stroke, a transthoracic echocardiogram (TTE) was pursued, which revealed a mobile mass on the anterior leaflet that prolapsed into the left atrium, along with nonspecific thickening of the posterior leaflet. A transesophageal echocardiogram (TEE) was performed to delineate further these lesions identified on the TTE, considering possibilities such as infective endocarditis, tumor, marantic endocarditis, and thrombus. The subsequent TEE identified two vegetations on the mitral valve; the largest measured 1.2 × 1.0 cm with a small perforation of the posterior leaflet (Figure 1a and Supporting Information 1: Video S1). The second vegetation measured 1.0 × 0.9 cm on the subvalvular apparatus of the anterior leaflet (Figure 1b and Supporting Information 2: Video S2). There was mild mitral regurgitation, and both the left atrium and left ventricle were of normal size. The patient remained afebrile throughout the hospital course, and two separate blood cultures did not yield any bacterial growth.

A thorough rheumatology workup was performed to rule out autoimmune etiologies. The rheumatology panel returned positive for antinuclear antibodies, anti-dsDNA antibodies, anti-histone antibodies, anticardiolipin antibodies, lupus anticoagulant, beta-2 glycoprotein IgM and IgA antibodies, silica clotting time, and diluted Russell viper venom time. These findings supported a diagnosis of SLE and secondary APS, and a diagnosis of LSE was established.

**Figure 2:**
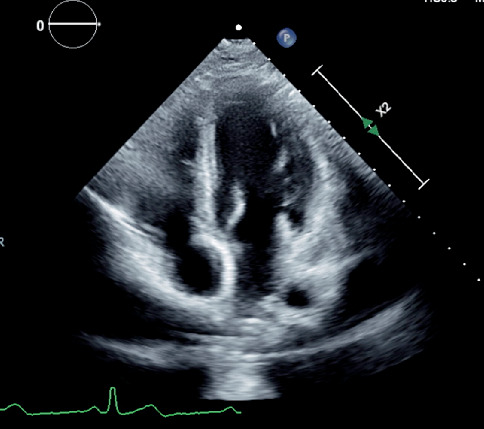
Transthoracic echocardiogram demonstrating the resolution of the previously observed vegetations.

Due to the size of the mitral valve lesions and the risk of recurrent emboli, a multidisciplinary team of cardiologists and cardiothoracic surgeons was assembled to assess the necessity for surgical intervention. Following extensive discussions among the multidisciplinary team, a repeat TTE was scheduled with the intention of proceeding to surgery if the vegetations persisted. The patient was started on anticoagulation with enoxaparin and gradually bridged to warfarin, aiming for an international normalized ratio goal of 2–3. Daily aspirin 81 mg and hydroxychloroquine (HCQ) 200 mg were initiated concurrently. Significant improvement in left-sided weakness was observed over the subsequent weeks, and a TTE conducted 4 weeks after the initial presentation demonstrated complete resolution of the vegetation (Figure 2).

## 3. Discussion

LSE, also known as marantic or verrucous endocarditis, is a noninfectious endocarditis commonly observed in SLE, APS, and certain solid tumors. Stroke is the most common presentation of LSE, with the aortic and mitral valves frequently involved [1, 2]. The underlying mechanism is ascribed to endothelial damage due to the deposition of inflammatory cells, complement, and immunoglobulin. The damaged endothelium predisposes the deposition of fibrin and platelets, increasing the risk of thromboembolic events and stroke [3]. Among the antibodies observed in SLE and APS, lupus anticoagulant and anticardiolipin antibodies are known independent risk factors for thrombosis [4, 5].

Treatment options for SLE-related endocarditis include anticoagulation [6] and HCQ [7]. The commonly used anticoagulants in LSE include warfarin and heparin, with close monitoring of the coagulation profile. HCQ, widely used in rheumatological diseases for its anti-inflammatory and antithrombotic properties, has been shown to reduce the risk of thromboembolic complications in patients with SLE [8, 9]. Our patient received 200 mg of HCQ and 5 mg of warfarin daily for their anti-inflammatory and anticoagulation effects, respectively. Due to the lack of robust randomized controlled trials (RCTs) assessing the efficacy of these therapies, current practices depend on limited reports and expert opinions.

Among the cases demonstrating the efficacy of these therapies for LSE, one describes a 40-year-old woman with aortic valve LSE that improved after treatment with HCQ, aspirin, and steroids [10]. While HCQ has been shown to have an anti-inflammatory role in LSE, the role of corticosteroids remains unclear and warrants further studies. Another case involved a 60-year-old woman with left-sided endocarditis secondary to LSE, who responded well to warfarin and immunosuppressants, resulting in the resolution of mitral valve vegetations after 6 months [11]. In contrast to this extended duration of 6 months, our patient showed resolution of the valvular lesions within 4 weeks of anticoagulation and anti-inflammatory therapy. Due to the absence of RCTs and society guidelines on LSE, there is no standard approach to these cases, particularly regarding treatment duration and agent selection [12].

The indications for surgical intervention in infective endocarditis are well defined and include heart failure, persistent infection, vegetation size larger than 10 mm, prosthetic valve endocarditis, and recurrent embolization despite the use of anticoagulants [13]. However, the criteria for valvular surgery remain ambiguous in cases of LSE and depend on a case-by-case assessment of risks and benefits. While surgical valve replacement with mechanical or tissue valves in APS has been performed successfully, it carries a risk of thromboembolic events and bleeding [14].

In this patient, multiple treatment modalities were considered following a personalized risk–benefit assessment and bleeding risk profile. The large size of the lesions and the presentation with a stroke necessitated operative management to prevent further complications associated with LSE. However, after careful consideration by the endocarditis team regarding the young age, high bleeding risk, and the documented success of conservative management, the patient was started on HCQ and warfarin, with a follow-up TTE scheduled before the valvular procedure. The follow-up TTE performed 4 weeks after the initial presentation showed complete resolution of the lesions, with no residual valve abnormalities. The patient demonstrated remarkable improvement in disease burden in less than a month, obviating the need for surgical management in this rare scenario.

## 4. Follow-Up

The patient was closely monitored in the general cardiology clinic and underwent repeat TTE at regular intervals to ensure that no new valvular lesions developed. The neurological weakness improved with pharmacotherapy and physical therapy, and the patient was continued on warfarin with routine monitoring in the coumadin clinic.

## 5. Conclusion

This case illustrates the successful conservative management of two large mitral valve vegetations. Since high-quality evidence on conservative management in LSE is limited, a multidisciplinary approach is recommended to assess treatment options. The duration of therapy required to demonstrate the resolution of lesions remains uncertain and needs to be individualized. However, additional studies are necessary to establish the optimal duration and dosage of treatment to standardize care. The establishment of an “endocarditis team,” as in the case of infective endocarditis, may help navigate the complex assessment of risks and benefits associated with each treatment approach. This could also reduce the necessity for surgical or invasive procedures, along with their associated complications.

## Data Availability

The data that support the findings of this study are available on request from the corresponding author. The data are not publicly available due to privacy or ethical restrictions.

## Supporting Information

Supporting Information 1 Video S1: Vegetation of size measuring 1.2 × 1.0 cm with a small perforation of the posterior leaflet.

Supporting Information 2 Video S2: The second vegetation measuring 1.0 × 0.9 cm on the subvalvular apparatus of the anterior leaflet.
